# Body image perception and body composition: assessment of perception inconsistency by a new index

**DOI:** 10.1186/s12967-019-02201-1

**Published:** 2020-01-14

**Authors:** Luciana Zaccagni, Natascia Rinaldo, Barbara Bramanti, Jessica Mongillo, Emanuela Gualdi-Russo

**Affiliations:** 1 Department of Biomedical Sciences and Surgical Specialties, Faculty of Medicine, Pharmacy and Prevention, University of Ferrara, Corso Ercole I d’Este 32, 44121 Ferrara, Italy; 2 Biomedical Sport Studies Center, University of Ferrara, Ferrara, Italy; 3 University Center for Studies on Gender Medicine, University of Ferrara, Ferrara, Italy

**Keywords:** Body image, Bioimpedance, Anthropometry, FID, FAI

## Abstract

**Background:**

A correct perception of the body image, as defined by comparison with actual anthropometric analyses, is crucial to ensure the best possible nutritional status of each individual. Bioimpedance analysis (BIA) represents a leading technique to assess body composition parameters and, in particular, the fat mass. This study examined the self-perception of body image at various levels of adiposity proposing a new index.

**Methods:**

We investigated 487 young Italian adults (mean age of males: 21.9 ± 2.4 years; mean age of females: 21.0 ± 2.2 years). Each subject could choose, on the Contour Drawing Rating Scale, the silhouette that he/she considered most resembling his/her perceived body image as well as his/her ideal body image. On each subject, we performed anthropometric measurements and determined the values of Fat mass and  %Fat with BIA. A new index, FAI^FAT^ (Feel fat status minus Actual fat status Inconsistency), was developed to evaluate possible fat status perception inconsistencies by BIA.

**Results:**

Based on ideal and feel body image comparison, women showed higher dissatisfaction than men and preferred slimmer silhouettes. FAI^FAT^ values indicated that the fat status perception was correct in the majority of the examined individuals and only three subjects showed a serious misperception.

**Conclusions:**

Our findings suggest that FAI^FAT^ is an appropriate index for assessing the perceived fat status from the body image when compared with data obtained by BIA. In a population, the use of this index will allow the correct identification of groups at risk for eating disorders.

## Background

An adequate nutritional status is essential to maintain healthy conditions in singular individuals and populations. Malnutrition impacts the risk of disease, the course of the disease, and enhances the risk of mortality [1–3]. The risk of morbidity and mortality increases particularly with increasing abdominal fat [4], while the body mass index (BMI) alone does not represent an adequate predictive indicator of the individual health status [5–7].

Obesity is increasing worldwide and, particularly, in industrialized countries [8]. In the same countries, there is an increase in negative perception of the body image and, consequently, an increase in eating disorders [9–13] and unhealthy behaviors. Recent studies have demonstrated an association between high BMI with body image dissatisfaction and self-reported overvaluation of the body size. This may lead to dietary concerns, depression and fear of weight gain [14, 15]. Other studies show that a poor body image perception leads to poor self-esteem with an increased risk of anxiety and depression [16, 17]. On the other hand, a self-image misperception, as well as a depressive status, boosts the risk of eating disorders [18, 19] and can lead to an unhealthy lifestyle, increasing sedentary and poor nutritional habits [20, 21]. All these factors can result in malnutrition with an increasing risk of disease and mortality [22]. In fact, strong discrepancies between the perceived and the ideal figure (dissatisfaction), as well as an incorrect self-image of the body size (inconsistency), can result in inappropriate behaviors, with serious and long-lasting implications on the health of the individuals [23]. Conversely, a positive body perception is commonly associated with self-esteem, optimism and a healthier nutritional behavior [24, 25].

Thus, to evaluate the nutritional status of an individual, we consider fundamental to explore his/her correct perception of the body image based on the body size evaluated through anthropometric methods. An objective evaluation of the actual body size, and in particular of the parameters of body composition, allows the individuals to undertake appropriate corrective actions in terms of diet and exercise, where necessary.

Body composition analysis can be carried out in adults with clinically available methods, such as dual-energy X-ray absorptiometry, Computed Tomography and Magnetic Resonance Imaging [26–29], yet anthropometric techniques are those most widely used for their reliability and simplicity of use.

Despite its utility in assessing conditions of malnutrition, which are of growing importance in modern societies, body image self-perception in relation to body size is still a poorly explored field. In 2014, Zaccagni et al. [30] developed a new tool (the FAI index) that assesses the perceived weight status, analyzing the figure chosen as their actual and BMI in a sample of undergraduate students. More recently, Cohen et al. [31] proposed an index (body weight self-satisfaction index) similar to the previous one on the basis of another Figure Rating Scale. For the present study, we decided to evaluate in a large sample of young adults the consistency of the body figure perceived as actual with the fat status objectively assessed by bioimpedance analysis (BIA). Although also this technique shows some few limits [32], BIA represents a leading method for body composition assessment and allows with confidence the estimation of fat percentage (%F) in obese individuals [29]. In addition, BIA is a more reliable anthropometric method for adiposity status assessment in comparison to BMI [33, 34].

Aim of this study was to propose a new index (FAI^FAT^), which relates the body image chosen as actual to body composition parameters (Fat) obtained with BIA. We took into consideration also possible differences of FAI^FAT^ between sexes. The FAI^FAT^ index gives a simple score to discriminate a subject according to own fat-status in underestimated, consistent or overestimated, allowing to promptly identify who needs corrective measures to solve any wrong nutritional behaviors.

## Methods

### Sample

We carried out a cross-sectional study on a sample of 487 Italian students in the Faculty of Medicine, Pharmacy and Prevention at the University of Ferrara (North-Italy) by convenience sampling selection. The students were 303 males (aged 21.9 ± 2.4) and 184 females (aged 21.0 ± 2.2).

The criteria for inclusion among the participants were: (1) being Italian; (2) being aged 18 years or older. Those with diagnosed health problems which may interfere with anthropometric measurements or body image perception were excluded.

The study protocol was approved by the Ethics Committee for Biomedical Research of the Ferrara University. After receiving explanations about the objectives of the study, the subjects of this survey provided written informed consent.

### Procedures

Stature and weight were measured according to standardized procedures [35] by trained operators with a mechanical scale (precision 0.1 kg, Seca) and a wall-mounted stadiometer (precision 0.1 cm; Magnimeter, Raven Equipment Limited, UK), respectively. BMI (weight/stature^2^, kg/m^2^) was calculated to define the weight status of the subjects. According to the WHO classification, BMI can be stratified into ‘underweight’, ‘normal weight’, ‘overweight’ or ‘obese’, encoded respectively as 1, 2, 3 and 4.

Body resistance (ohm) and reactance (ohm) values were taken for each subject by means of an Akern 101 Sport Edition analyzer (Akern, Florence, Italy), with a right-sided tetrapolar electrode-placement in standard conditions. Bioelectrical values were used to assess body composition parameters, i.e. fat free mass (FFM, kg), fat mass (FM, kg), and fat percentage (%F). FFM was calculated with the regression equation proposed by Kyle et al. [36]. FM was calculated as weight—FFM and %F as (FM/weight) * 100.

On the basis of %F and cut offs by sex and age proposed by Gallagher et al. [37], the subjects were classified into ‘underfat’, ‘normal fat’, ‘overfat’ and ‘very overfat’ categories, respectively encoded as 1, 2, 3 and 4.

Body image perception was assessed by means of the Contour Drawing Rating Scale [38]. For each sex, nine silhouettes were proposed, numbered and sorted in ascending order, from emaciated (silhouette 1) to obese (silhouette 9). Each subject had the possibility to choose the silhouettes closest to his/her own perception (Feel figure) and to his/her own ideal (Ideal figure) body shape. Dissatisfaction in body image perception was calculated as feel–ideal difference (FID) [39, 40].

The inconsistency between the body image perception (Feel figure) and the actual weight status assessed by means of BMI was calculated as FAI (feel weight status minus actual weight status inconsistency) [30].

To assess the inconsistency on the basis of body fat assessment by BIA and the feel figure, we devised the index FAI^FAT^ (Feel fat status minus actual fat status Inconsistency by BIA). FAI^FAT^ uses the silhouette matching technique as a proxy to verify whether there is a realistic fat status perception in the subject. The FAI^FAT^ was computed by subtracting the conventional code assigned to the actual fat status of the subject (code: 1 for underfat, 2 for normal fat, 3 for overfat and 4 for very overfat, as assessed by BIA) from the one corresponding to her/his feel figure according to the following correspondence: silhouettes 1 and 2 match fat status 1 (underfat); silhouettes 3, 4 and 5 match fat status 2 (normal fat); silhouettes 6 and 7 match fat status 3 (overfat); silhouettes 8 and 9 match fat status 4 (very overfat).

The FAI^FAT^ scores range from − 3 to + 3: negative FAI^FAT^ values point to an underestimated fat status, whereas positive FAI^FAT^ values to an overestimated fat status. A FAI^FAT^ score of 0 means a consistent perception of the own fat status.

### Statistical analyses

Distribution normality was assessed by sex (Kolmogorov–Smirnov test). Comparisons between sexes were performed using the t-test (for traits normally distributed) or U Mann–Whitney test. Comparisons between fat status categories was performed using Kruskal–Wallis non parametric test (for traits not normally distributed) and a Tukey’s post hoc test was used for comparisons among groups. Comparisons between dependent samples were carried out with Wilcoxon test. Categorical data were analyzed by means of Pearson’s Chi square test. Comparisons among fat status categories were performed by means of Kruskal–Wallis test. Spearman’s rank correlation was used to evaluate associations between %F and the new index FAI^FAT^. Next, linear regression analysis was performed and visually inspected in order to identify risk values of body misperception.

Values of p < 0.05 were considered statistically significant. All statistical analyses were performed using the Statistica software, version 11.0 (StatSoft srl, Tulsa, OK).

## Results

In Table 1, we summarized the mean anthropometric values and the mean body image indicators derived from the sample separately by sex. Regarding anthropometric and body composition parameters, females were significantly shorter and lighter than males, with significantly lower mean values of BMI and FFM, and significantly higher mean values of adiposity parameters (FM and %F). The silhouettes chosen by males were, on average, significantly bigger than those chosen by females both in term of Feel and Ideal figures. Females reported a significantly higher FID value than males, which demonstrated in young women a higher dissatisfaction due to their wish to be slimmer than they actually were. In any case, both sexes preferred an Ideal figure which was significantly thinner than their own (*p *= 0.0401 in males; *p *< 0.0001 in females). Nevertheless, both sexes demonstrated a good perception of their body, as revealed by the values close to 0 of the FAI and FAI^FAT^ indices, although females showed a slight tendency to see themselves as fatter (positive FAI^FAT^ values) and males as thinner (negative FAI^FAT^ values) than they actually were (Table 1). When we made a comparison between the mean FAI and FAI^FAT^ values within the two groups, the differences resulted significant (FAI vs FAI^FAT^: *p *< 0.0001 in males; *p *= 0.0147 in females).

**Table 1:** Anthropometric characteristics, body image perception, weight-status and fat-status by sex

Traits	Males, n = 303	Females, n = 184	p
Stature (cm)	178.3 ± 6.9	163.7 ± 6.2	< 0.0001^a^
Weight (kg)	75.6 ± 10.4	59.5 ± 8.3	< 0.0001^a^
BMI (kg/m^2^)	23.8 ± 2.8	22.2 ± 2.8	< 0.0001^a^
FFM (kg)	60.7 ± 6.6	41.5 ± 5.5	< 0.0001^a^
FM (kg)	14.8 ± 5.6	17.2 ± 5.0	< 0.0001^a^
F %	19.1 ± 4.9	28.7 ± 5.3	< 0.0001^a^
Feel figure	5.3 ± 1.1	5.0 ± 1.2	0.0030^b^
Ideal figure	5.2 ± 0.5	3.9 ± 0.9	< 0.0001^b^
FID	0.13 ± 1.07	1.09 ± 0.98	< 0.0001^b^
FAI	0.19 ± 0.54	0.25 ± 0.55	0.3226^b^
FAI^FAT^	− 0.06 ± 0.60	0.15 ± 0.58	0.0004^b^
Weight status	n (%)	n (%)	< 0.0001^c^
Underweight	5 (1.7%)	16 (8.9%)	
Normal weight	221 (73.7%)	145 (80.6%)	
Overweight	64 (21.3%)	15 (8.3%)	
Obese	10 (3.3%)	4 (2.2%)	
Fat status	n (%)	n (%)	< 0.0001^c^
Underfat	3 (1.0%)	15 (8.8%)	
Normal fat	167 (57.4%)	122 (71.8%)	
Overfat	89 (30.6%)	28 (16.5%)	
Very overfat	32 (11.0%)	5 (2.9%)	

^a^Student's t-test

^b^Kruskal–Wallis non-parametric test

^c^Chi-Squared test

In Table 1, we also reported the absolute and relative frequencies of weight- and fat-status found in our sample, divided by sex. The differences between sexes were significant in both weight- and fat-status percentages. All post hoc group-wise comparisons, apart from obese, in weight status were significant (p < 0.01). Among the males, we observed a higher percentage of overweight, overfat and very overfat subjects, while among females a higher number of under- and normal weight, underfat and normal fat.

Table 2 shows the mean results of the body image perception scores divided by sex and by fat status categories. In both sexes, the mean Feel figures significantly differ between the different categories, increasing in value as the %F increases. In contrast, the mean Ideal figure chosen is similar for all categories in males, while its score significantly increases in value within the fat categories in females. Mean FID values increase with increasing body fat, both in males and females. However, females reported lower dissatisfaction than males in the under-fat groups, but higher in the other fat categories and all of them wished to be thinner, while under fat and normal fat males preferred a higher number in the body image rating scale. The mean FAI values, that indicate the consistent perception of themselves on the basis of BMI, are significantly different within the fat categories only in males. However, in both sexes and in all categories with the exception of very overfat females, the mean FAI values are positive (indicating an overestimation of their own weight-status), or close to 0 (indicating a general consistent perception of themselves). Moreover, the difference in FAI values is significant only between normal fat and overfat males (p = 0.0106). In fact, while overfat males had higher FAI values, the other fat categories had values of FAI very close to each other, indicating a similar perception of their fat.

**Table 2:** Body image perception by sex and fat-status categories

Traits	Underfat, Mean ± SD	Normal fat, Mean ± SD	Overfat, Mean ± SD	Very overfat, Mean ± SD	
Males	n = 3	n = 165	n = 89	n = 32	p^a^
Feel figure	4.00 ± 0.00	4.81 ± 0.97	5.81 ± 0.84	6.64 ± 0.72	< 0.0001
Ideal figure	5.00 ± 0.00	5.13 ± 0.55	5.24 ± 0.50	5.31 ± 0.53	0.2104
FID	− 1.00 ± 0.00	− 0.32 ± 0.93	0.57 ± 0.84	1.33 ± 0.88	< 0.0001
FAI	0.00 ± 0.00	0.13 ± 0.51	0.35 ± 0.55	0.12 ± 0.71	0.0153
FAI^FAT^	1.00 ± 0.00	0.22 ± 0.45	− 0.29 ± 0.46	− 0.97 ± 0.40	< 0.0001
Females	n = 15	n = 122	n = 28	n = 5	p^a^
Feel figure	3.87 ± 1.25	4.83 ± 0.99	5.77 ± 0.88	7.20 ± 0.45	< 0.0001
Ideal figure	3.40 ± 1.18	3.82 ± 0.80	4.13 ± 0.88	5.20 ± 0.84	0.0018
FID	0.47 ± 1.55	1.01 ± 0.86	1.64 ± 0.61	2.00 ± 0.71	0.0001
FAI	0.07 ± 0.70	0.30 ± 0.50	0.29 ± 0.53	− 0.40 ± 0.89	0.0764
FAI^FAT^	0.80 ± 0.41	0.23 ± 0.46	− 0.43 ± 0.50	− 0.80 ± 0.45	< 0.0001

^a^Kruskal–Wallis non-parametric test

When considering the FAI^FAT^, the mean values are significantly different between the fat categories with the highest values (both positive and negative) in the outermost groups (positive in underfat and negative in very overfat), indicating a higher inconsistency between actual body fat and the Feel figure. Compared with FAI, the FAI^FAT^ values resulted significantly different (Wilcoxon test; p < 0.05) in all the fat groups and in both sexes, except in underfat males and in very overfat females (Table 2).

The majority of the male students chose the silhouettes 5 and 6 (Table 3) to define their perceived body image (feel figure), whereas their mean BMI and F% fall within the range of normal weight and normal fat. In fact, as demonstrated by the mean FAI and FAI^FAT^ values, they generally overestimated their body size and underestimated their body fat. The female students chose prevalently silhouette number 4 and 5 (Table 3) as their perceived body image (Feel figure), which is in accordance with their mean weight- and fat-status. In general, females tended to fat overestimation (FAI^FAT^ values > 0), whereas males to fat underestimation (FAI^FAT^ values < 0).

**Table 3:** Percentage of feel and ideal figures selected by males and females separately and mean BMI, %F, FAI index and FAI^FAT^ index of subjects that chose each silhouette as their feel

Silhouette number	Feel, n (%)	Ideal, n (%)	BMI, Mean ± SD	%F, Mean ± SD	FAI, Mean ± SD	FAI^FAT^, Mean ± SD
Males						
1	0 (0.0)	0 (0.0)	–	–	–	–
2	2 (0.7)	0 (0.0)	18.5 ± 0.9	12.2 ± 1.1	− 0.50 ± 0.70	− 1.00 ± 0.00
3	7 (2.3)	2 (0.7)	20.9 ± 2.0	16.4 ± 4.1	0.14 ± 0.38	− 0.14 ± 0.38
4	65 (21.7)	11 (3.7)	21.2 ± 1.6	15.8 ± 4.0	0.05 ± 0.21	− 0.03 ± 0.35
5	91 (30.3)	215 (72.1)	23.4 ± 1.7	17.7 ± 3.5	− 0.12 ± 0.36	− 0.29 ± 0.50
6	89 (29.7)	68 (22.8)	24.5 ± 2.1	20.9 ± 3.8	0.63 ± 0.53	0.25 ± 0.64
7	42 (14.0)	2 (0.7)	27.7 ± 2.5	24.1 ± 4.6	0.14 ± 0.61	− 0.24 ± 0.73
8	4 (1.3)	0 (0.0)	30.7 ± 4.6	33.2 ± 6.8	0.50 ± 0.58	0.00 ± 0.00
9	0 (0.0)	0 (0.0)	–	–	–	–
Females						
1	0 (0.0)	1 (0.5)	–	–	–	–
2	5 (2.8)	5 (2.7)	18.3 ± 1.1	19.3 ± 6.2	− 0.60 ± 0.55	− 0.25 ± 0.50
3	10 (5.6)	55 (30.2)	18.5 ± 1.2	23.7 ± 4.9	0.70 ± 0.48	0.30 ± 0.48
4	43 (23.9)	76 (41.8)	20.5 ± 1.6	25.9 ± 4.8	0.16 ± 0.37	0.02 ± 0.27
5	69 (38.3)	40 (22.0)	22.1 ± 2.0	28.1 ± 5.2	0.03 ± 0.24	− 0.06 ± 0.51
6	36 (20.0)	6 (3.3)	23.6 ± 1.5	30.7 ± 3.3	0.82 ± 0.38	0.71 ± 0.46
7	17 (9.4)	0 (0.0)	25.6 ± 3.0	33.4 ± 7.7	0.33 ± 0.77	0.00 ± 0.73
8	0 (0.0)	0 (0.0)	–	–	–	–
9	0 (0.0)	0 (0.0)	–	–	–	–

This aspect has been analysed more in detail in Table 4, which shows the frequencies of the subjects, divided per fat-status and sex, that underestimated (FAI^FAT^ < 0), overestimated (FAI^FAT^ > 0) or had the right perception (FAI^FAT^ = 0) of their own body fat. Most males demonstrated a right perception of their body, especially those with normal fat and overfat status. The three subjects in the category “underfat” overestimated their body fat, while the majority of the very overfat subjects underestimated it. Also, the majority of females had a good perception of their body, even if 24% of them tended to overestimate it and just 10% to underestimate it. In particular, the majority of normal fat females had a right perception of their body, meanwhile underfat females saw themselves fatter and very overfat females saw themselves thinner than they were.

**Table 4:** Body image perception inconsistency by sex and fat-status categories

Fat categories	FAI^FAT^ < 0 Underestimation, n (%)	FAI^FAT^ = 0 Correct perception, n (%)	FAI^FAT^ > 0 Overestimation, n (%)
Males			
Underfat	0 (0.0)	0 (0.0)	3 (100)
Normal fat	2 (1.6)	124 (97.6)	1 (0.8)
Overfat	26 (28.9)	64 (71.1)	0 (0.0)
Very overfat	29 (90.6)	3 (9.4)	0 (0.0)
Total	57 (22.6)	191 (75.8)	4 (1.6)
Females			
Underfat	0 (0.0)	3 (20.0)	12 (80.0)
Normal fat	1 (0.8)	92 (75.4)	29 (23.8)
Overfat	12 (42.9)	16 (57.1)	0 (0.0)
Very overfat	4 (66.7)	2 (33.3)	0 (0.0)
Total	17 (9.9)	113 (66.1)	41 (24.0)

In general, the higher percentage of students who underestimated their body fat is among overfat subjects of both sexes (males: 45.6%; females: 70.6%) and very overfat male subjects (50.9%). Notably, among those who overestimated their body fat, there were 75.0% of underfat males and 70.7% of normal fat females (Table 4).

**Fig. 1:**
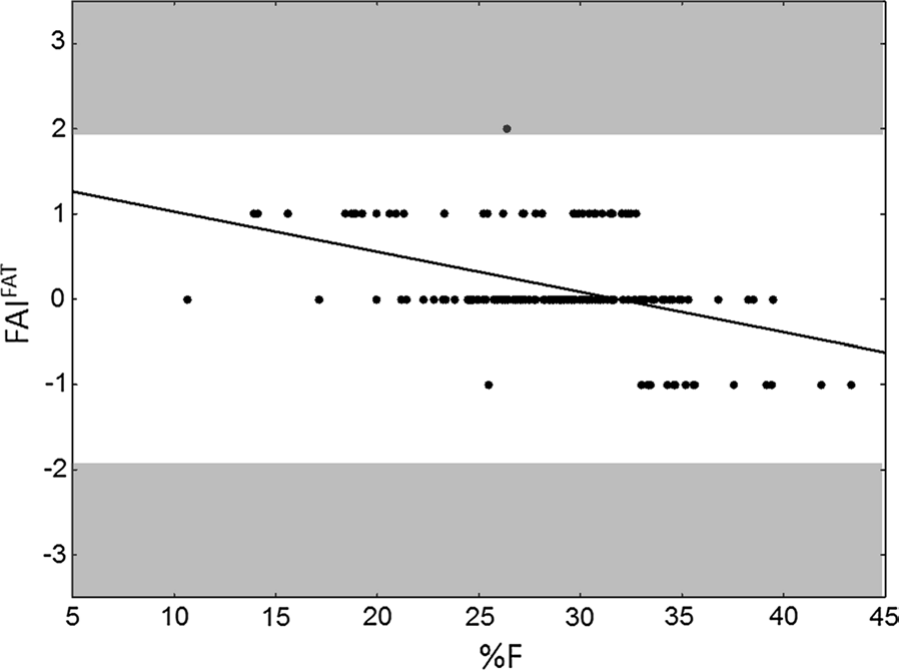
Scatterplot representing the relationship between %F and FAI^FAT^ in females. Highlighted in grey the two risk zones

**Fig. 2:**
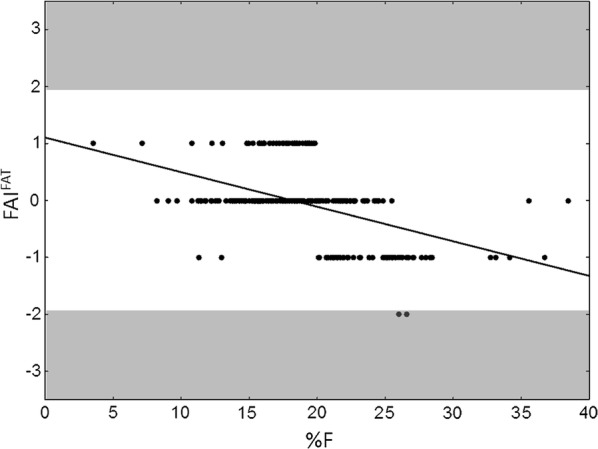
Scatterplot representing the relationship between %F and FAI^FAT^ in males. Highlighted in grey the two risk zones

Given the highly significant negative correlations between FAI^FAT^ and %F in both sexes of the examined sample (males: r = − 0.5013; females: r = − 0.3564; *p *< 0.0001), we performed a regression analysis identifying body misperception for subjects with FAI^FAT^ ≥ 2 or ≤ − 2 (Figs. 1 and 2) in accordance with previous studies [41, 42] on the interpretation of perceived figure (Feel figure) and actual anthropometric values. In particular, only one subject from the female subsample (Fig. 1) had FAI^FAT^ = 2: this normal fat young woman misperceived her body as very overfat (FAI^FAT^ = Feel fat status 4—actual fat status 2 = 2). In the male subsample (Fig. 2), there were two overfat subjects with FAI^FAT^ = − 2, misperceiving their body as normal fat (FAI^FAT^ = Feel fat status 2—actual fat status 4 = − 2).

## Discussion

In this study, we examined the body composition and the body image perception of a sample of Italian University students and we proposed a new index, FAI^FAT^, in order to evaluate the inconsistency between the perceived body image and the measured fat status.

Our findings on this sample, in which both sexes are well represented, suggest that the body image perception, used as a proxy and measured by the fat status in relation to the estimated body image, was adequate in most subjects. With the exception of three individuals over the entire sample (0.6%), all the considered subjects had a consistent perception of their body with a general tendency to fat overestimation in females and to fat underestimation in males. In particular, according to the new proposed index, under-fat students of both sexes overestimated their body fat and over-fat students underestimated their body fat, suggesting that individuals from extreme groups poorly assess their body fat.

More in general and in accordance with literature in the field [11, 12, 43], the analysis of body image perception shows that females were higher dissatisfied than males and preferred slimmer silhouettes than males did. Almost 23% of males chose silhouette 6 (representing overweight/overfat) as their ideal. The reason might be that they misunderstood this silhouette interpreting it as a more muscular body image, as reported also by other studies [44].

This new index (FAI^FAT^) and the other one previously proposed (FAI) [30] assess the inconsistency between the body image perception and the actual size of an individual. Nevertheless, the first (FAI^FAT^) evaluates the inconsistency on the basis of fat status (%F), while the latter on the basis of weight status (BMI). In the present study, the %F was derived from the analysis of bioelectric impedance (BIA). While BMI tends to overestimate subjects with a high level of fat-free mass [45], the fat status does not seem to be affected by the same limitations and can be applied even on athletes. A further development of this study will consider applying FAI ^FAT^ to body composition parameters obtained using different methodologies (e.g., plicometry). The bias between FAI and FAI^FAT^ is confirmed by our results with FAI showing almost all positive values, and FAI^FAT^ positive values (indicating overestimation) prevalent in lower fat categories and negative values (indicating underestimation) in the overfat and obese categories.

## Conclusions

Our new proposed index contributes to the literature a proxy measure of general appropriateness of body image perception according to fat status. Since this index is based on the fat component of the body, its analysis implies that interventions on eating disorders could be more effective by simultaneously monitoring the evolution of body composition and body perception of the patients. This approach might achieve greater success in combating eating disorders.

In conclusion, we deem that further research into health risk is necessary and urgent, especially with regard to non-communicable diseases [46, 47]. At a population level, the assessment of body perception and composition by FAI^FAT^ ensures an easy identification of sub-groups in risk zones with the view to monitor and correct their health situation. This control strategy is particularly important to avoid health risk behaviors in case of under fat and over fat people misperceiving their fat status.

## Data Availability

The data of this study are not publicly available, but they are available from the corresponding authors upon reasonable request.

## Abbreviations

BIA: bio-impedance analysis
BMI: body mass index
FAI: feel weight status minus actual weight status inconsistency
FAI^FAT^: feel fat status minus actual fat status inconsistency assessed by BIA
FFM: fat free mass
FID: feel ideal difference
FM: fat mass
%F: fat percentage
SD: standard deviation

## References

[1] Allison DB, Faith MS, Heo M, Kotler DP (1997). Hypothesis concerning the U-shaped relation between body mass index and mortality. Am J Epidemiol.

[2] Bigaard J, Frederiksen K, Tjønneland A, Thomsen BL, Overvad K, Heitmann BL (2004). Body fat and fat-free mass and all-cause mortality. Obes Res.

[3] Reis JP, Macera CA, Araneta MR, Lindsay SP, Marshall SJ, Wingard DL (2009). Comparison of overall obesity and body fat distribution in predicting risk of mortality. Obesity.

[4] Mathieu P, Pibarot P, Larose É, Poirier P, Marette A, Després J-P (2008). Visceral obesity and the heart. Int J Biochem Cell Biol.

[5] Gualdi-Russo E, Zaccagni L, Dallari GV, Toselli S (2015). Anthropometric parameters in relation to glycaemic status and lipid profile in a multi-ethnic sample in Italy. Public Health Nutr.

[6] Ashwell M, Gunn P, Gibson S (2012). Waist-to-height ratio is a better screening tool than waist circumference and BMI for adult cardiometabolic risk factors: systematic review and meta-analysis. Obes Rev.

[7] Maffetone PB, Rivera-Dominguez I, Laursen PB (2016). Overfat and underfat: new terms and definitions long overdue. Front public Heal..

[8] Bixby H, Bentham J, Zhou B, Di Cesare M, Paciorek CJ (2019). Rising rural body-mass index is the main driver of the global obesity epidemic in adults. Nature.

[9] Grabe S, Ward LM, Hyde JS (2008). The role of the media in body image concerns among women: a meta-analysis of experimental and correlational studies. Psychol Bull.

[10] Swami V, Frederick DA, Aavik T, Alcalay L, Allik J, Anderson D (2010). The attractive female body weight and female body dissatisfaction in 26 countries across 10 world regions: results of the International Body Project I. Personal Soc Psychol Bull..

[11] Toselli S, Rinaldo N, Gualdi-Russo E (2016). Body image perception of African immigrants in Europe. Global Health..

[12] Gualdi-Russo E, Rinaldo N, Khyatti M, Lakhoua C, Toselli S (2016). Weight status, fatness and body image perception of North African immigrant women in Italy. Public Health Nutr..

[13] Toselli S, Rinaldo N, Gualdi-Russo E (2019). Length of residence and obesity risk among North African immigrant women in Italy. Econ Hum Biol..

[14] Grilo CM, Ivezaj V, Lydecker JA, White MA (2019). Toward an understanding of the distinctiveness of body-image constructs in persons categorized with overweight/obesity, bulimia nervosa, and binge-eating disorder. J Psychosom Res.

[15] Paans NPG, Bot M, Brouwer IA, Visser M, Penninx BWJH (2018). Contributions of depression and body mass index to body image. J Psychiatr Res.

[16] Paxton SJ, Neumark-Sztainer D, Hannan PJ, Eisenberg ME (2006). Body dissatisfaction prospectively predicts depressive mood and low self-esteem in adolescent girls and boys. J Clin child Adolesc Psychol..

[17] Wilson RE, Latner JD, Hayashi K (2013). More than just body weight: the role of body image in psychological and physical functioning. Body Image.

[18] Goldschmidt AB, Wall MM, Loth KA, Neumark-Sztainer D (2015). Risk factors for disordered eating in overweight adolescents and young adults. J Pediatr Psychol.

[19] Toselli S, Rinaldo N, Caccialupi MG, Gualdi-Russo E (2018). Psychosocial indicators in north african immigrant women in Italy. J Immigr Minor Heal..

[20] Lee E-Y, Myre M, Hwang J, Chun H, Seo E, Pabayo R (2017). Body weight misperception and psychological distress among young South Korean adults: the role of physical activity. Glob Heal Res Policy..

[21] Buscemi S, Marventano S, Castellano S, Nolfo F, Rametta S, Giorgianni G (2018). Role of anthropometric factors, self-perception, and diet on weight misperception among young adolescents: a cross-sectional study. Eat Weight Disord.

[22] Abdelaal M, le Roux CW, Docherty NG (2017). Morbidity and mortality associated with obesity. Ann Transl Med..

[23] Duncan DT, Wolin KY, Scharoun-Lee M, Ding EL, Warner ET, Bennett GG (2011). Does perception equal reality? Weight misperception in relation to weight-related attitudes and behaviors among overweight and obese US adults. Int J Behav Nutr Phys Act..

[24] Avalos L, Tylka TL, Wood-Barcalow N (2005). The body appreciation scale: development and psychometric evaluation. Body Image..

[25] Rinaldo N, Zaccagni L, Gualdi-Russo E (2016). Soccer training programme improved the body composition of pre-adolescent boys and increased their satisfaction with their body image. Acta Paediatr.

[26] Goodpaster BH, Thaete FL, Kelley DE (2000). Composition of skeletal muscle evaluated with computed tomography. Ann N Y Acad Sci.

[27] Williams JE, Wells JCK, Wilson CM, Haroun D, Lucas A, Fewtrell MS (2006). Evaluation of Lunar Prodigy dual-energy X-ray absorptiometry for assessing body composition in healthy persons and patients by comparison with the criterion 4-component model. Am J Clin Nutr.

[28] Duren DL, Sherwood RJ, Czerwinski SA, Lee M, Choh AC, Siervogel RM (2008). Body composition methods: comparisons and interpretation. J Diabetes Sci Technol.

[29] Lee D-H, Park K, Ahn S, Ku E, Jung K, Kim Y (2015). Comparison of abdominal visceral adipose tissue area measured by computed tomography with that estimated by bioelectrical impedance analysis method in Korean subjects. Nutrients.

[30] Zaccagni L, Masotti S, Donati R, Mazzoni G, Gualdi-Russo E (2014). Body image and weight perceptions in relation to actual measurements by means of a new index and level of physical activity in Italian university students. J Transl Med..

[31] Cohen E, Gradidge PJ-L, Micklesfield LK, Norris SA (2019). Relationship between body mass index and body image disturbances among South African mothers and their daughters living in Soweto, Johannesburg. Fam Commun Health.

[32] Gualdi-Russo E, Toselli S (2002). Influence of various factors on the measurement of multifrequency bioimpedance. Homo.

[33] Ibáñez ME, Mereu E, Buffa R, Gualdi-Russo E, Zaccagni L, Cossu S (2015). New specific bioelectrical impedance vector reference values for assessing body composition in the Italian–Spanish young adult population. Am J Hum Biol..

[34] Zaccagni L, Rinaldo N, Bramanti B, Gualdi-Russo E (2017). Relation between lifestyle behaviors and body composition patterns among healthy young Italians: a cross-sectional study. J Sports Med Phys Fitness.

[35] Weiner JS, Lourie JA (1981). Practical human biology.

[36] Kyle UG, Genton L, Karsegard L, Slosman DO, Pichard C (2001). Single prediction equation for bioelectrical impedance analysis in adults aged 20–94 years. Nutrition.

[37] Gallagher D, Heymsfield SB, Heo M, Jebb SA, Murgatroyd PR, Sakamoto Y (2000). Healthy percentage body fat ranges: an approach for developing guidelines based on body mass index. Am J Clin Nutr.

[38] Thompson MA, Gray JJ (1995). Development and validation of a new body-image assessment scale. J Pers Assess.

[39] Mciza Z, Goedecke JH, Steyn NP, Charlton K, Puoane T, Meltzer S (2005). Development and validation of instruments measuring body image and body weight dissatisfaction in South African mothers and their daughters. Public Health Nutr..

[40] Mchiza ZJ, Goedecke JH, Lambert EV (2011). Intra-familial and ethnic effects on attitudinal and perceptual body image: a cohort of South African mother-daughter dyads. BMC Public Health..

[41] Gualdi-Russo E, Albertini A, Argnani L, Celenza F, Nicolucci M, Toselli S (2008). Weight status and body image perception in Italian children. J Hum Nutr Diet.

[42] Gualdi-Russo E, Manzon VS, Masotti S, Toselli S, Albertini A, Celenza F (2012). Weight status and perception of body image in children: the effect of maternal immigrant status. Nutr J.

[43] Brennan MA, Lalonde CE, Bain JL (2017). Body image perceptions: do gender differences exist?. Psi Chi J Psychol Res..

[44] Jones DC, Bain N, King S (2008). Weight and muscularity concerns as longitudinal predictors of body image among early adolescent boys: a test of the dual pathways model. Body Image.

[45] Romero-Corral A, Somers VK, Sierra-Johnson J, Thomas RJ, Collazo-Clavell ML, Korinek J (2008). Accuracy of body mass index in diagnosing obesity in the adult general population. Int J Obes (Lond)..

[46] Gillen MM (2015). Associations between positive body image and indicators of men’s and women’s mental and physical health. Body Image.

[47] NCD Countdown 2030 (2018). Worldwide trends in non-communicable disease mortality and progress towards Sustainable Development Goal target 34. Lancet (London, England)..

